# β-Cyclodextrin-Polyacrylamide Hydrogel for Removal of Organic Micropollutants from Water

**DOI:** 10.3390/molecules26165031

**Published:** 2021-08-19

**Authors:** Xia Song, Nana Nyarko Mensah, Yuting Wen, Jingling Zhu, Zhongxing Zhang, Wui Siew Tan, Xinwei Chen, Jun Li

**Affiliations:** 1 Department of Biomedical Engineering, Faculty of Engineering, National University of Singapore, 7 Engineering Drive 1, Singapore 117574, Singapore; a0045788@u.nus.edu (X.S.); nn.mensah@u.nus.edu (N.N.M.); bieweny@nus.edu.sg (Y.W.); erizhuj@nus.edu.sg (J.Z.); biezhozh@nus.edu.sg (Z.Z.); 2 Institute of Materials Research and Engineering, A*STAR (Agency for Science, Technology and Research), 2 Fusionopolis Way, Singapore 138634, Singapore; wuisiew@gmail.com (W.S.T.); CHEN_Xinwei@nrf.gov.sg (X.C.)

**Keywords:** β-cyclodextrin, inclusion complex, polyacrylamide, hydrogel, organic micropollutant removal

## Abstract

Water pollution by various toxic substances remains a serious environmental problem, especially the occurrence of organic micropollutants including endocrine disruptors, pharmaceutical pollutants and naphthol pollutants. Adsorption process has been an effective method for pollutant removal in wastewater treatment. However, the thermal regeneration process for the most widely used activated carbon is costly and energy-consuming. Therefore, there has been an increasing need to develop alternative low-cost and effective adsorption materials for pollutant removal. Herein, β-cyclodextrin (β-CD), a cheap and versatile material, was modified with methacrylate groups by reacting with methacryloyl chloride, giving an average degree of substitution of 3 per β-CD molecule. β-CD-methacrylate, which could function as a crosslinker, was then copolymerized with acrylamide monomer via free-radical copolymerization to form β-CD-polyacrylamide (β-CD-PAAm) hydrogel. Interestingly, in the structure of the β-CD-PAAm hydrogel, β-CD is not only a functional unit binding pollutant molecules through inclusion complexation, but also a structural unit crosslinking PAAm leading to the formation of the hydrogel 3D networks. Morphological studies showed that β-CD-PAAm gel had larger pore size than the control PAAm gel, which was synthesized using conventional crosslinker instead of β-CD-methacrylate. This was consistent with the higher swelling ratio of β-CD-PAAm gel than that of PAAm gel (29.4 vs. 12.7). In the kinetic adsorption studies, phenolphthalein, a model dye, and bisphenol A, propranolol hydrochloride, and 2-naphthol were used as model pollutants from different classes. The adsorption data for β-CD-PAAm gel fitted well into the pseudo-second-order model. In addition, the thermodynamic studies revealed that β-CD-PAAm gel was able to effectively adsorb the different dye and pollutants at various concentrations, while the control PAAm gel had very low adsorption, confirming that the pollutant removal was due to the inclusion complexation between β-CD units and pollutant molecules. The adsorption isotherms of the different dye and pollutants by the β-CD-PAAm gel fitted well into the Langmuir model. Furthermore, the β-CD-PAAm gel could be easily recycled by soaking in methanol and reused without compromising its performance for five consecutive adsorption/desorption cycles. Therefore, the β-CD-PAAm gel, which combines the advantage of an easy-to-handle hydrogel platform and the effectiveness of adsorption by β-CD units, could be a promising pollutant removal system for wastewater treatment applications.

## 1. Introduction

Water pollution by various toxic substances including heavy metals, dye molecules and aromatic compounds remains a serious environmental problem [1]. Among the different pollutants, the occurrence of organic micropollutants, which include anthropogenic pollutants from pharmaceuticals, pesticides, personal care products, steroids and industrial chemicals, has become a worldwide environmental problem [2]. One example of endocrine disrupting compounds is bisphenol A (BPA), a component of plastics from industrial origin and found present in surface water [3]. Pharmaceutical pollutants include propranolol (PR), a β-blocker used to regulate blood pressure in hypertension treatment, and it was found in sewage treatment plant effluents as well as in streams and rivers [4]. 2-Naphthol (2-NO), a model naphthol pollutant, was also present in the environment from the chemical, pesticides, paper and painting manufactory, and it is also known to be toxic and harmful to humans and environment [5]. To address these issues, there has been an increasing effort to develop technologies to remove these pollutants in wastewater treatment [6,7].

In addition to the conventional primary and secondary treatment processes employed by wastewater treatment plants, advanced treatments for micropollutant removal have been developed, such as adsorption by activated carbon, ozonation and advanced oxidation processes, and membrane processes [2]. Among these, adsorption process, which is effective in removing specific micropollutants, has low production of toxic by-products and is relatively less expensive than membrane processes and advanced oxidation processes [8,9]. However, the main disadvantage is the costly and energy-consuming thermal regeneration process for activated carbons, which are the most widely used adsorbents for pollutants removal [10]. Regenerating activated carbons requires heating to very high temperatures, ~800–850 °C, and does not fully restore the performance [11,12]. Therefore, research into development of different regeneration processes, such as chemical regenerations, or alternative low-cost adsorbents has been undertaken [8,9,10,12,13].

β-Cyclodextrin (β-CD), which is a cyclic oligosaccharide consisting of α-1,4-linked 7 D(+)-glucose units, has been extensively studied for its ability to form supramolecular inclusion complexes with a variety of molecules that could fit into the hydrophobic cavity of β-CD [14,15,16,17,18,19,20,21]. Due to this unique property, there have been increasing interests and investigations to analyze the complexations between β-CD and various organic pollutant molecules, including BPA, PR·HCl and 2-NO [22,23,24,25]. In addition, different dye molecules, such as phenolphthalein (Php), have also been used as models to study and gain insights for the inclusion complexes formed by β-CD and the dye molecules [26,27]. To extend its applications to pollutant removal in wastewater treatment, the β-CD component has to be in the form of insoluble adsorbent materials. With abundant hydroxyl groups, β-CD could be easily modified or crosslinked into insoluble polymers, using crosslinkers such as epichlorohydrin or tetrafluoroterephthalonitrile, developing into various β-CD-based polymer adsorbents [1,28,29,30,31,32,33,34,35]. Alsbaiee et al. prepared a porous polymer of β-CD with high surface area by using a rigid aromatic crosslinker [28]. A recent study investigated a β-CD-based polymer using both epichlorohydrin and tetrafluoroterephthalonitrile as crosslinkers to develop a multifunctional adsorbent system [36]. β-CD-conjugated nanocomposites have also been investigated. One example is graphene oxide-β-CD nanocomposite for BPA removal [37]. A β-CD-functionalized silica composite was also developed for the removal of steroid residues from water [38]. However, despite the high adsorption efficiency for the pollutants, these insoluble adsorbent materials generally need to be filtered during handling, which might be a concern during applications.

Hydrogel is a three-dimensional network that consists of crosslinked hydrophilic polymers and can retain large amount of water, leading to swelling of the macromolecular structure in aqueous solutions. It has a wide range of applications across different fields including biological, medical and environmental areas [39,40]. β-CD-containing hydrogels have also been developed for pollutant adsorption and removal applications [41,42,43]. Polyacrylamide (PAAm)-based polymers are widely used as a flocculant in water and wastewater treatment, a soil conditioner in agricultural applications, or a viscosity enhancer and friction reducer for enhanced oil recovery and high volume hydraulic fracturing [44]. PAAm could also be crosslinked to form hydrogels with very low toxicity, good stability and ability to swell in water, making them suitable for broad applications in biology, medicine and agriculture [45,46]. Therefore, PAAm hydrogel is an excellent candidate as a platform for incorporating β-CD units for pollutant removal.

In this work, to combine the advantage of a PAAm hydrogel for easy handling and the effective pollutant adsorption ability of β-CD, a robust pollutant removal system has been developed. In the structure of the β-CD-PAAm hydrogel, β-CD is not only a functional unit binding pollutant molecules through inclusion complexation, but also a structural unit crosslinking PAAm leading to the formation of the hydrogel 3D networks. β-CD was firstly modified with multiple methacrylate groups using methacryloyl chloride. Then, β-CD with multiple methacrylate groups was used as a crosslinker and copolymerized with acrylamide monomer via free-radical polymerization to form β-CD-polyacrylamide (β-CD-PAAm) hydrogel. The morphologies and swelling properties of β-CD-PAAm gel were analyzed and compared with PAAm gel without β-CD component as a control. The effectiveness of β-CD-PAAm gel in pollutant removal was demonstrated and evaluated in the kinetic and thermodynamic adsorption studies using various model dye and pollutants. The regeneration properties and performance of β-CD-PAAm gel was also investigated.

## 2. Experimental Section

### 2.1. Materials

β-Cyclodextrin (β-CD, ≥98%) was obtained from Tokyo Chemical Industry and dried under vacuum at 100 °C for one day before usage. Methacryloyl chloride (MA, >90.0%) was purchased from Tokyo Chemical Industry. Triethylamine (TEA, ≥99%) was purchased from Merck. Acrylamide (AAm, ≥99%), *N*,*N*’-methylenebisacrylamide (MBA, 99%), *N*,*N*,*N*’,*N*’-tetramethylethylenediamine (TEMED, ≥99%), and ammonium persulfate (APS, 98%) were obtained from Sigma-Aldrich. Phenolphthalein (Php, ACS reagent), bisphenol A (BPA, ≥99%), propranolol hydrochloride (PR∙HCl, ≥99%), and 2-napthol (2-NO, 99%) were purchased from Sigma-Aldrich. All solvents were purchased from VWR.

### 2.2. Synthesis of β-Cyclodextrin-Methacrylate (β-CD-MA)

The modification of β-CD with methacrylate groups using methacryloyl chloride was adapted from the reported protocol [47,48,49]. Generally, dried β-CD (3.9 g, 3.4 mmol) was dissolved in 30 mL of degassed anhydrous *N*,*N*-dimethylformamide (DMF), followed by the addition of anhydrous TEA (3.4 mL, 24.4 mmol). The reaction mixture was cooled down to 0 °C while stirring in an ice bath. Methacryloyl chloride (2.0 mL, 20.5 mmol) in 1.5 mL of anhydrous DMF was added dropwise into the mixture while stirring. The mixture was allowed to slowly increase to room temperature. After stirring for 4 h, triethylamine hydrochloride was filtered, and the clear mixture solution was precipitated in 300 mL of acetone. The precipitate collected by centrifugation was redissolved in 5 mL of DMF and precipitated in 50 mL of acetone. The solid product was further purified by column chromatography using a solvent mixture of 1-propanol-water-ammonium hydroxide-toluene (6:3:1:1) as eluent. The solvent was then evaporated under vacuum to obtain the pure solid product, denoted as β-CD-MA. Yield: 2.4 g, 52%. FTIR (KBr): ν = 1717 (ν(C=O)), 1638 cm^−1^ (ν(C=C)); ^1^H NMR (600 MHz, DMSO-d_6_): δ 1.84 (m, methyl protons of methacrylate group); 3.20–4.60 (m, H-2–H-6 and OH-6 of β-CD); 4.83 (b, H-1 of β-CD); 5.50–6.10 (m, vinyl protons of methacrylate group, overlapped with OH-2 and OH-3 of β-CD).

### 2.3. Synthesis of β-Cyclodextrin-Polyacrylamide (β-CD-PAAm) Gel

β-CD-PAAm gel was synthesized by copolymerizing AAm monomers and β-CD-MA via free-radical polymerization initiated by a redox pair of APS and TEMED. The protocol was optimized from the reported literature [50]. β-CD-MA, which had three methacrylate groups on one β-CD, could act as crosslinkers in the gel formation. Therefore, no additional crosslinkers were added. In a typical example, β-CD-MA (3.24 g, 2.42 mmol) and AAm (11.25 g, 158.3 mmol) were dissolved in a mixture of DMSO and water (1:1) to prepare 100 mL of solution in a volumetric flask. Then, 2 mL of the prepared solution was mixed with 50 µL of 10% (*w*/*v*) APS and 10 µL of TEMED, and vortexed for a few seconds. The prepolymer solution (400 µL) was dispensed into each well of a 24-well cell culture plate and allowed to polymerize for 30 min. After that, the 24-well plates containing the synthesized hydrogels were submerged into deionized (DI) water. The unreacted monomers were removed from the hydrogels by soaking in DI water for 5 days. During purification in DI water, the disk-shape hydrogels came out of the wells and became fully swollen. The purified hydrogels were then lyophilized to obtain the dry hydrogel product. Yield: 42.0 mg per piece of hydrogel disk, 72.5%.

### 2.4. Synthesis of Polyacrylamide (PAAm) Gel

PAAm gel with MBA as the crosslinkers instead of β-CD-MA was synthesized as a control gel, following the same procedures for producing β-CD-PAAm gel. As MBA has two double bonds per molecule while β-CD-MA has three, the amount of double bonds was kept the same for the synthesis of β-CD-PAAm gel and PAAm gel. In brief, MBA (0.563 g, 3.65 mmol) and AAm (11.25 g, 158.3 mmol) were dissolved in a mixture of DMSO and water (1:1) to prepare 100 mL of solution in a volumetric flask. Then, 2 mL of the prepared solution were mixed with 50 µL of 10% (*w*/*v*) APS and 10 µL of TEMED, and vortexed for a few seconds. The prepolymer solution (400 µL) was dispensed into each well of a 24-well cell culture plate and allowed to polymerize for 30 min. After that, the 24-well plates containing the synthesized hydrogels were submerged into DI water. The unreacted monomers were removed from the hydrogels by soaking in DI water for 5 days. During purification in DI water, the disk-shape hydrogels came out of the wells and became fully swollen. The purified hydrogels were then lyophilized to obtain the dry hydrogel product. Yield: 43.1 mg per piece of hydrogel disk, 91.2%.

### 2.5. Characterizations

^1^H nuclear magnetic resonance (NMR) spectra were recorded on a Varian VNMRS 600 MHz NMR spectrometer at room temperature. Chemical shifts were referenced to the solvent peak (δ = 2.50 ppm for DMSO-d_6_).

Fourier transform infrared (FTIR) spectra of samples in potassium bromide (KBr) were measured on a Shimadzu IRPrestige-21 spectrometer in the region of 4000–500 cm^−1^.

UV-Vis measurement was performed with a TECAN Infinite M200 PRO microplate reader. Absorbance of 150 µL of the sample solution was measured at 552 nm, 276 nm, 290 nm and 273 nm for Php, BPA, PR·HCl and 2-NO, respectively.

Scanning electron microscopy (SEM) images of PAAm gel, β-CD-PAAm gel, and β-CD-PAAm gel after adsorption of BPA were taken with a Hitachi FlexSEM 1000 scanning electron microscope at 5 kV. The surface and cross-sectional morphologies of lyophilized hydrogels were studied. The lyophilized hydrogels were cut with a scalpel to obtain the cross-sections.

X-ray photoelectron spectroscopy (XPS) spectra were recorded with a Kratos Axis Ultra DLD X-ray photoelectron spectrophotometer equipped with an Al Kα X-ray source (1486.69 eV).

### 2.6. Swelling Studies

The fully swollen β-CD-PAAm gel and PAAm gel (5 replicates each) were removed from DI water, blot-dried to remove excess water and weighed. The hydrogels were then lyophilized. The dried gels were weighed again. The swelling ratio of each hydrogel was calculated using the following Equation (1):

(1) $$\mathrm{Swelling}\;\mathrm{ratio}=\frac{W_{s}-W_{d}}{W_{d}}$$

where W_s_ (mg) and W_d_ (mg) are the weight of the swollen and dried hydrogels, respectively.

### 2.7. Kinetic Studies of Pollutant Removal

Kinetic studies of pollutant removal by β-CD-PAAm gel and PAAm gel were conducted for Php, BPA, PR·HCl and 2-NO at room temperature (25 °C). Php was dissolved in sodium bicarbonate buffer (0.1 M, pH 10.5) and the other pollutants were dissolved in DI water. Dried β-CD-PAAm gel or PAAm gel was immersed in 10 mL of 0.1 mM solution of each pollutant in a 20 mL glass vial and stirred. At each predetermined time point, 150 µL of the solution were taken and its absorbance was measured with a microplate reader (Infinite M200 PRO, TECAN). A series of pollutant solutions with varying concentrations were prepared to produce a calibration curve by measuring the absorbance at 552 nm, 276 nm, 290 nm and 273 nm for Php, BPA, PR·HCl and 2-NO, respectively. The residual concentration of the pollutant solution at each time point was determined using the calibration curve. The amount of pollutant uptake and percentage removal of pollutant was calculated.

The kinetics data for pollutant adsorption was fitted into pseudo-second-order model with the following expression (2):

(2) $$\frac{t}{q_{t}}=\;\frac{1}{k_{2}q_{e}^{2}}+\;\frac{t}{q_{e}}$$

where q_t_ and q_e_ are the adsorption capacity (mg of pollutant per g of hydrogel) at time t (min) and at equilibrium, respectively, and k_2_ is the second-order rate constant (g mg^−1^ min^−1^).

### 2.8. Thermodynamic Studies of Pollutant Removal

Thermodynamic studies of pollutant removal by β-CD-PAAm gel and PAAm gel were carried out for Php, BPA, PR·HCl and 2-NO at room temperature (25 °C). A series of pollutant solutions with varying concentrations were prepared for Php (0.025–0.5 mM), BPA (0.05–1.0 mM), PR·HCl (0.035–1.0 mM) and 2-NO (0.035–1.0 mM). Dried β-CD-PAAm gel or PAAm gel was immersed in 10 mL of each pollutant solution with different concentrations in a 20 mL glass vial and stirred. After reaching equilibrium, 150 µL of the solution were taken and its absorbance was measured with a microplate reader (Infinite M200 PRO, TECAN). The residual concentration of the pollutant solution was determined using the calibration curve.

The adsorption data was fitted into the Langmuir isotherm model, as expressed in the following Equation (3):

(3) $$\frac{1}{q_{e}}=\frac{1}{q_{\max,e}}+\frac{1}{q_{\max,e}{\mathrm{KC}}_{e}}$$

where q_e_ is the adsorption capacity (mg of pollutant per g of hydrogel) at equilibrium, q_max,e_ is the maximum adsorption capacity at equilibrium, C_e_ (mmol L^−1^) is the residual concentration of pollutant at equilibrium, and K is the adsorption equilibrium constant (M^−1^).

### 2.9. Hydrogel Recycling Studies

β-CD-PAAm gel was immersed in 10 mL of 0.1 mM BPA solution in a 20 mL glass vial and stirred at room temperature (25 °C). After reaching equilibrium, the percentage removal of BPA was estimated. β-CD-PAAm gel was then regenerated by immersing in 10 mL of methanol and stirring overnight. After removing the methanol solution, the regenerated β-CD-PAAm gel was dried under vacuum and ready for use for the next cycle. The adsorption/desorption process was conducted five times.

## 3. Results and Discussion

### 3.1. Synthesis of β-Cyclodextrin-Methacrylate

**Figure 1:**
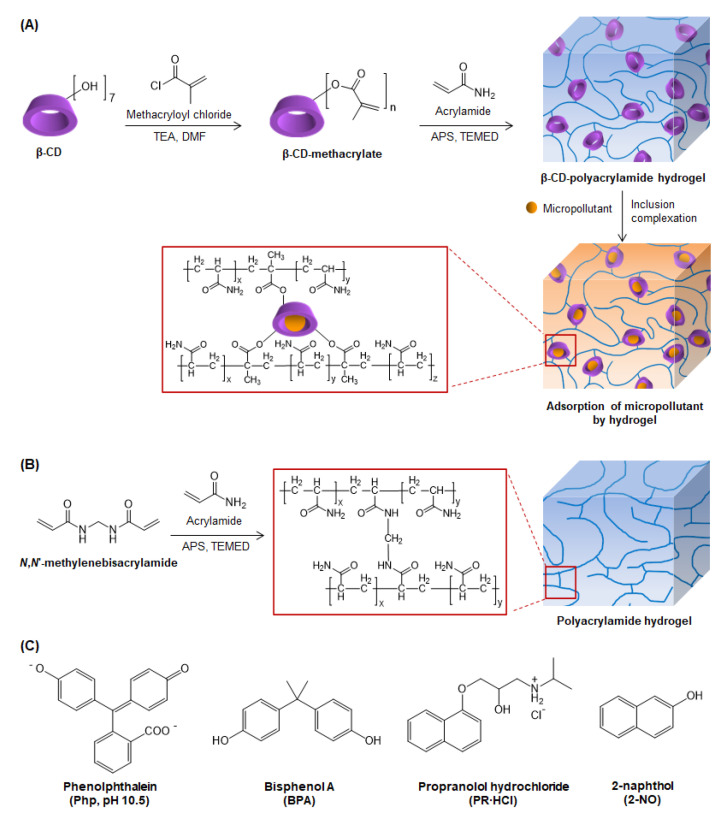
(**A**) Synthesis scheme for β-cyclodextrin methacrylate (β-CD-MA) and β-CD-polyacrylamide (β-CD-PAAm) hydrogel, and micropollutant removal via inclusion complexation between β-CD in the hydrogel and micropollutant molecules. (**B**) Synthesis scheme for polyacrylamide (PAAm) hydrogel using *N*,*N*’-methylenebisacrylamide as crosslinker. (**C**) Chemical structures of model dye and pollutants tested.

β-CD-MA was synthesized according to the protocol described in Figure 1A, which was adapted from the reported literature [47,48]. β-CD was modified with methacrylate groups using methacryloyl chloride. The successful conjugation of methacrylate groups to β-CD was confirmed by ^1^H NMR measured in DMSO-d_6_, as shown in Figure S1. The signals from both β-CD and the methacrylate groups have been observed for β-CD-MA in Figure S1B, such as signals for H-7 of methacrylate group around 1.84 ppm and H-8 and H-9 of methacrylate group overlapping with OH-2 and OH-3 of β-CD around 5.50–6.10 ppm. As compared with pure β-CD, the peaks for β-CD-MA were broadened. This might be due to the restriction of the molecular motions by the modifications. According to the literature and our previous study [38,51], the primary hydroxyl groups of β-CD at the 6-position are more nucleophilic than the secondary hydroxyl groups and more subjected to the modifications. Therefore, the methacrylate groups are more likely to be bonded to β-CD’s 6-positioned hydroxyl groups. By comparing the integrations of the signals for methyl protons of methacrylate groups (CH_2_=C(C*H*_3_)-, around 1.84 ppm) to those for the 1-positioned protons of β-CD around 4.83 ppm, the degree of substitution was estimated to be 3. As the β-CD was modified with multiple methacrylate groups, it could function as crosslinkers in the subsequent hydrogel formation without the addition of other conventional crosslinkers such as MBA crosslinker.

FTIR measurement was also carried out to further confirm the conjugation of methacrylate groups onto β-CD. Figure S2 shows the FTIR spectra of pure β-CD and β-CD-MA. Compared to pure β-CD, the β-CD-MA showed an appearance of a new peak at around 1717 cm^−1^ due to the C=O stretching of the ester bond, and an increase of the peak at around 1638 cm^−1^, which is characteristic of C=C stretching [52]. These confirmed the successful conjugation of methacrylate groups onto β-CD.

### 3.2. Synthesis and Characterizations of Hydrogels

**Figure 2:**
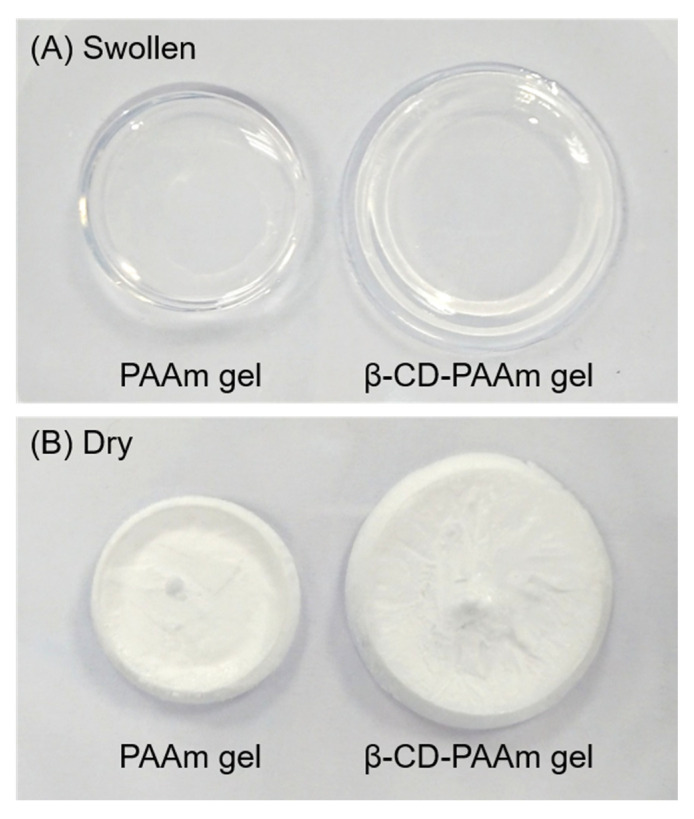
Photographs of PAAm gel (left) and β-CD-PAAm gel (right) in the swollen state (**A**) and dry state (**B**).

β-CD-MA was incorporated into PAAm hydrogel by copolymerizing with AAm monomers and crosslinking PAAm polymers to form β-CD-PAAm hydrogel (Figure 1A). PAAm gel crosslinked with MBA was also synthesized in the absence of β-CD-MA as a control (Figure 1B). After purification in DI water, the purified PAAm gel and β-CD-PAAm gel became fully swollen to different extents (Figure 2A). Both disk-shape hydrogels were then lyophilized for further analysis (Figure 2B).

**Figure 3:**
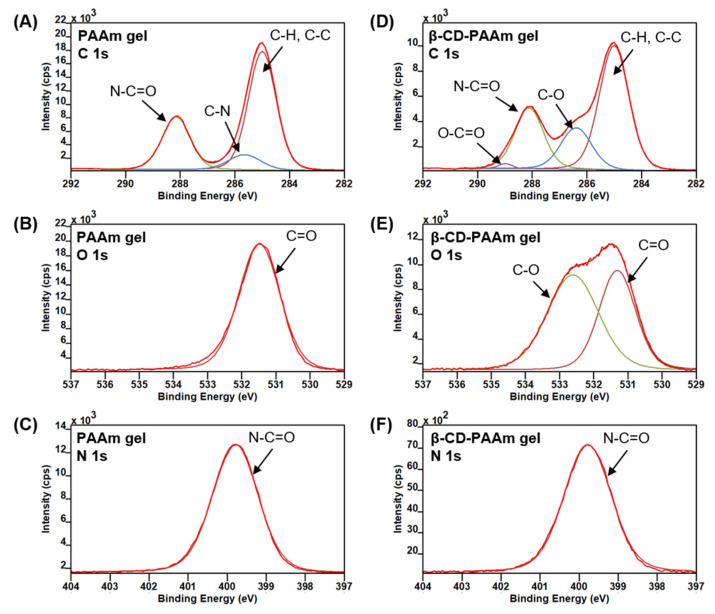
High-resolution XPS spectra of (**A**) C 1s, (**B**) O 1s, and (**C**) N 1s peaks of PAAm gel. High-resolution XPS spectra of (**D**) C 1s, (**E**) O 1s, and (**F**) N 1s peaks of β-CD-PAAm gel.

To confirm the incorporation of β-CD component into the hydrogel, the surface chemical composition of the PAAm gel and β-CD-PAAm gel was analysed by XPS (Figure 3). Figure 3A,D shows the high-resolution XPS spectra of C 1s peaks for PAAm gel and β-CD-PAAm gel, respectively. The peak for C 1s of PAAm gel can be deconvoluted into three peaks at 285.0, 285.7 and 288.2 eV, which can be assigned to C-H/C-C, C-N and N-C=O, respectively [53]. On the other hand, the peak for C 1s of β-CD-PAAm gel can be deconvoluted into four peaks at 285.0, 286.4, 288.1 and 289.0 eV, corresponding to C-H/C-C, C-O, N-C=O and O-C=O, respectively [54]. In addition, the O 1s spectrum of PAAm gel shows only one C=O peak at 531.5 eV in Figure 3B, whereas the O 1s peak of β-CD-PAAm gel can be deconvoluted into C=O peak at 531.3 eV and C-O peak at 532.6 eV (Figure 3E). These data strongly supported the presence of β-CD component in the β-CD-PAAm gel. For both PAAm gel and β-CD-PAAm gel, there is only one N 1s peak at 399.8 eV, which can be assigned to N-C=O (Figure 3C,F).

The XPS survey spectra of these two hydrogels are shown in Figure S3. The atomic concentrations (%) of the elements C, O and N for the hydrogels are shown in Table 1. The atomic ratios of N/C and N/O for PAAm gel are 0.261 and 1.004, respectively. However, the N/C and N/O ratios for β-CD-PAAm gel are 0.211 and 0.617, respectively. The increase of atomic concentration of O and decrease of atomic concentration of N confirmed the incorporation of the β-CD component into the β-CD-PAAm gel.

**Figure 4:**
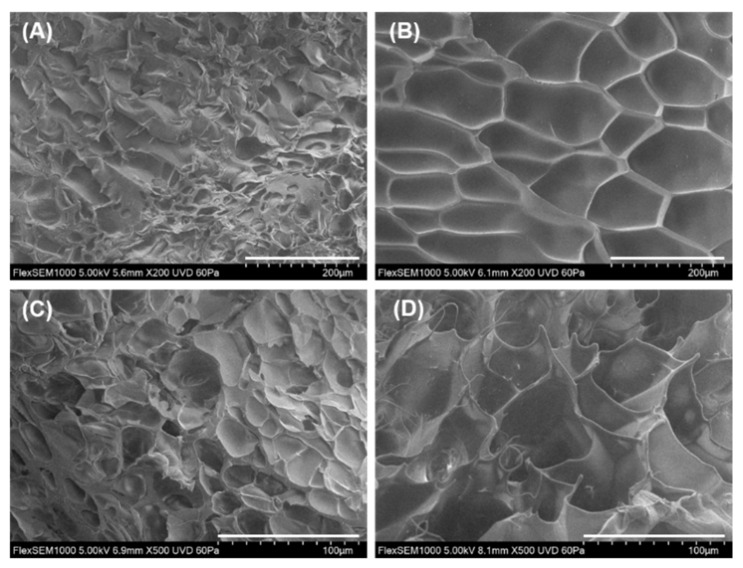
SEM images showing surface morphologies of lyophilized PAAm gel (**A**) and β-CD-PAAm gel (**B**) (scale bar = 200 µm). SEM cross-sectional images of PAAm gel (**C**) and β-CD-PAAm gel (**D**) (scale bar = 100 µm).

The morphologies of PAAm gel and β-CD-PAAm gel were studied by SEM. Figure 4 shows the surface morphologies and cross-sectional morphologies of the two lyophilized hydrogels. The lyophilized hydrogels were cut with a scalpel to obtain the cross-sections. It can be observed that the β-CD-PAAm gel has larger pores than the PAAm gel. The larger pore size of β-CD-PAAm gel may contribute to its higher swelling ratio as compared to PAAm gel.

After hydrogel synthesis, the hydrogels were purified by soaking in DI water for 5 days. During the purification process, the hydrogels became fully swollen to different extents. The weight of the swollen hydrogels and the lyophilized hydrogels were taken and compared. Table 2 summarizes the weight of the swollen and dried hydrogels and the swelling ratios. It was observed that β-CD-PAAm gel’s swelling ratio is 2.3 times of that of PAAm gel, presumably attributed to the larger pore size of β-CD-PAAm gel. This may facilitate the diffusion of the pollutants into the hydrogels and the subsequent inclusion complexation with β-CD units.

### 3.3. Kinetic Studies of Pollutant Removal

The pollutant removal properties of β-CD-PAAm gel were evaluated in kinetic studies using 10 mL of 0.1 mM of a model dye, Php, and three model pollutants, BPA, PR·HCl and 2-NO. PAAm gel was also tested as a control. Php, a known pH indicator, gives a pink color in basic solutions and changes to colorless lactonoid dianion when complexing with β-CD [55]. BPA, a component of plastics, is an endocrine disruptor [3]. PR represents a pollutant from pharmaceuticals as a β-blocker in hypertension treatment [4]. 2-NO is a model naphthol pollutant [5]. The concentrations of these model dye and pollutants were monitored by measuring the absorbance at 552 nm, 276 nm, 290 nm and 273 nm, respectively.

**Figure 5:**
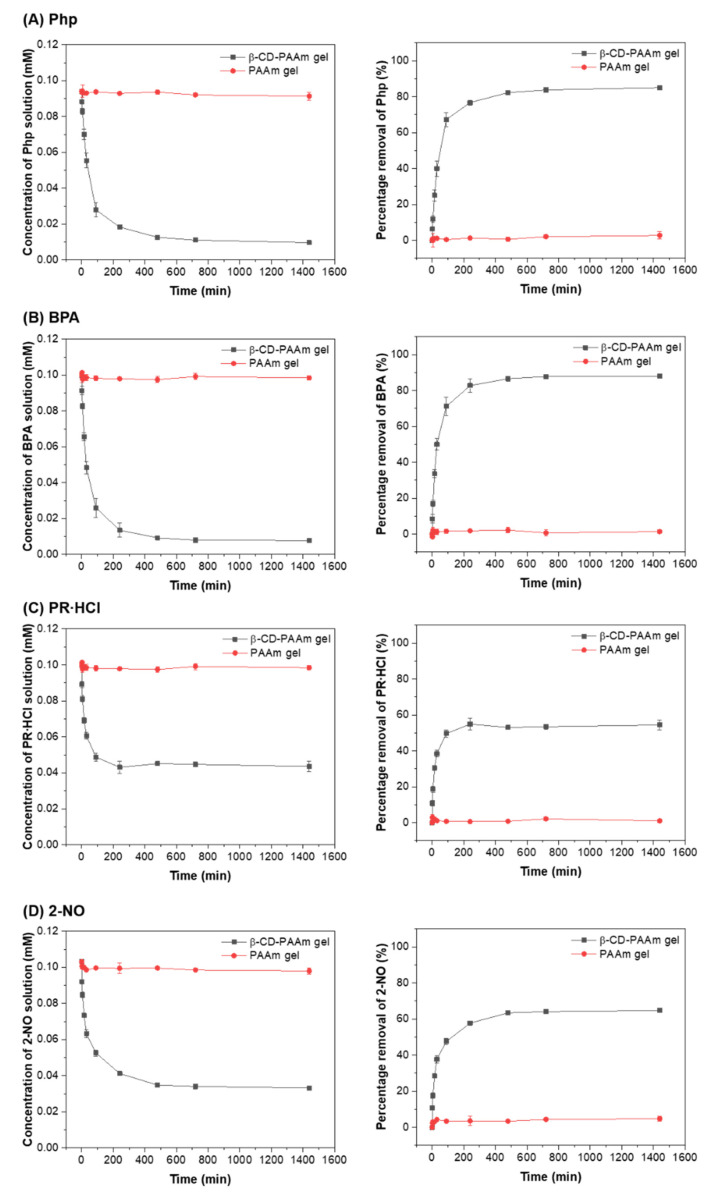
Graphs showing changes of pollutant concentrations (left) and percentage removal of pollutant (right) over time for (**A**) Php, (**B**) BPA, (**C**) PR∙HCl and (**D**) 2-NO solutions upon contact with β-CD-PAAm gels and PAAm gels. Data represent mean ± S.D. (*n* = 3).

Figure 5 shows the change of concentrations of each pollutant with time upon contact with the dried hydrogels. The cumulative percentage removal was also calculated. The dried β-CD-PAAm gel started to swell and adsorb pollutants when added to 10 mL of pollutant solutions. The adsorption reached equilibrium after about 8 h. The β-CD-PAAm gel took longer time to reach equilibrium than the reported studies [28,29], probably because the β-CD-PAAm gel had a higher swelling ratio and the pollutant solution needed to diffuse in to complex with the β-CD units. It was also observed that the equilibrium pollutant uptake was the highest for BPA (88%), followed by Php (87%). The uptake was lower for 2-NO (60%) and PR·HCl (54%). This might be because the association constant between β-CD and BPA is similar to that between β-CD and Php, and both are higher than those between β-CD and 2-NO, and between β-CD and PR·HCl [22,23,24,25,26,27].

In comparison, PAAm gel without β-CD units could not adsorb the dye or the pollutants even after a long time. This further confirms that the pollutant removal was due to the inclusion complexation between the β-CD units inside the β-CD-PAAm gel and the dye or pollutant molecules.

**Figure 6:**
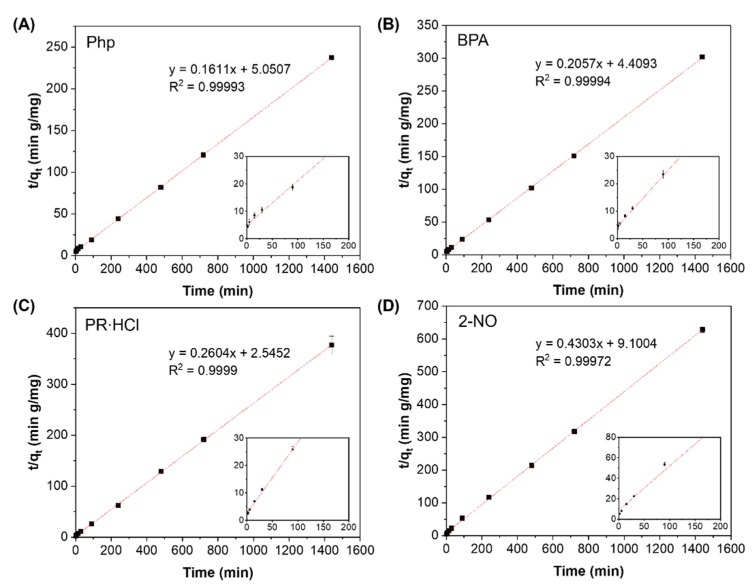
Linear fittings to pseudo-second-order kinetic model for adsorption of (**A**) Php, (**B**) BPA, (**C**) PR∙HCl and (**D**) 2-NO by β-CD-PAAm gels. Data represent mean ± S.D. (*n* = 3). The insets are the corresponding enlarged scale of the linear fittings for the first 200 min.

The kinetics data for β-CD-PAAm gel fitted well into the pseudo-second-order model with all the correlation coefficient R^2^ around 0.9997–0.9999 (Figure 6). The rate constant k_2_ and the q_e_ values are summarized in Table 3. The calculated q_e_ values are in good consistency with the experimental values. These calculated values for BPA adsorption were smaller than the reported values [28,29]. This might be because those systems are non-hydrogel-based and the major component was β-CD. In addition, q_e_ was calculated as mg of pollutant adsorbed per g of hydrogel. If q_e_ was calculated as mg of pollutant adsorbed per g of β-CD component (feed ratio: 9.4 mg of β-CD unit per 42 mg of hydrogel), the q_e_ value would be increased to 21.72 mg/g, which is comparable to the reported studies, in which the major component of the adsorbent was β-CD [28,29].

It should be noted that the ester groups in β-CD-PAAm gel hydrolyzed in the basic solution of Php (pH 10.5) after stirring for a long time. However, the β-CD units could still complex with Php, causing the color change. The decrease of absorbance at 552 nm indicates that the amount of free or uncomplexed Php has decreased in the solution. This is because Php gives a pink color in basic solutions and changes to colorless lactonoid dianion when complexing with β-CD. Therefore, the data are still useful for understanding the adsorption behavior of β-CD units.

### 3.4. Thermodynamic Studies of Pollutant Removal

**Figure 7:**
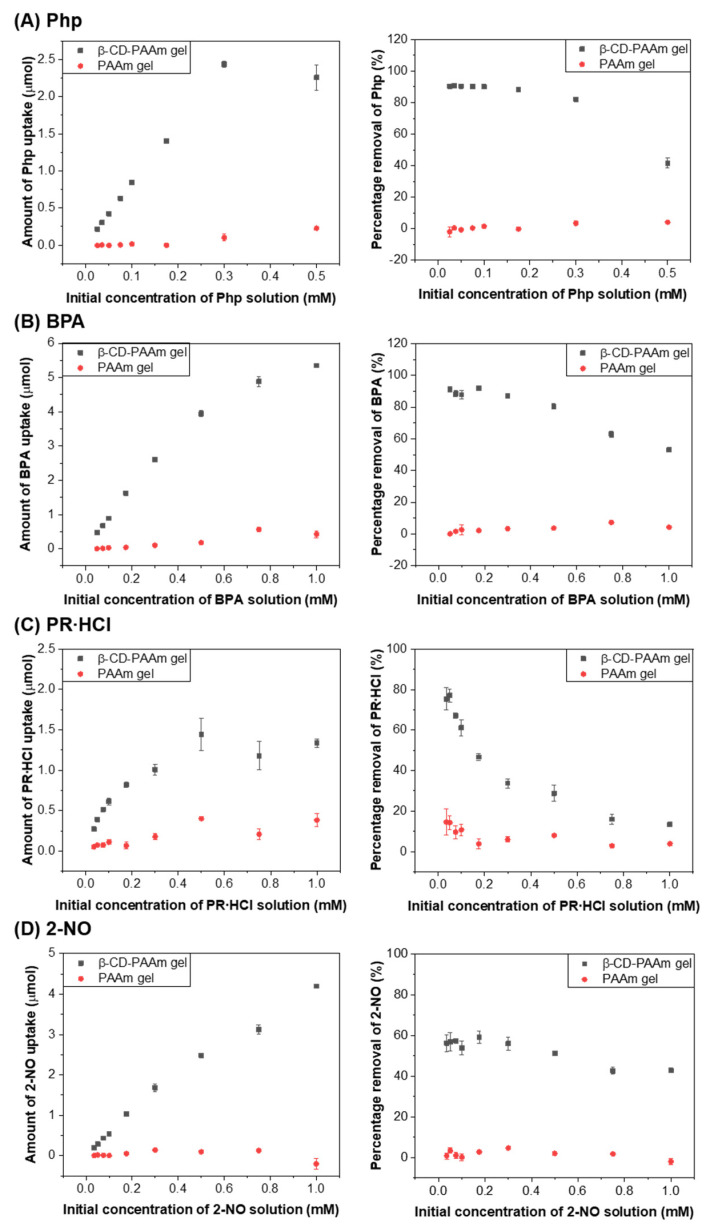
Graphs showing amount of pollutant uptake (left) and the percentage removal of pollutant (right) as a function of initial pollutant concentration for (**A**) Php, (**B**) BPA, (**C**) PR∙HCl and (**D**) 2-NO by β-CD-PAAm gels and PAAm gels. Data represent mean ± S.D. (*n* = 3).

The adsorption behavior was further analyzed by using different concentrations of the dye and pollutant solutions for β-CD-PAAm gel and PAAm gel. The hydrogels were immersed in the solutions until equilibrium was reached. The amount of uptake and percentage removal was plotted against the initial concentrations of pollutants, as shown in Figure 7. The amount of uptake for each pollutant increased with increasing initial pollutant concentrations by the β-CD-PAAm gel. On the other hand, the adsorption of the pollutants remained low for PAAm gel at various initial concentrations, due to the absence of β-CD units.

**Figure 8:**
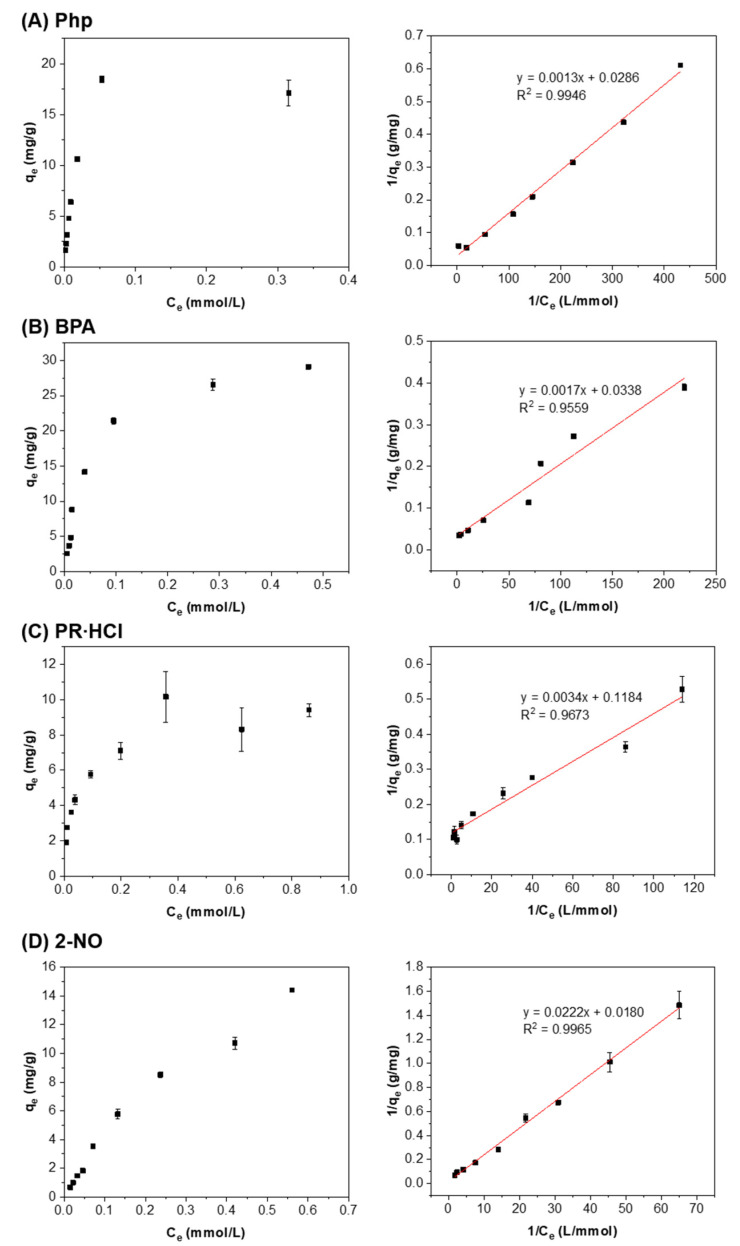
The q_e_ values as a function of pollutant equilibrium concentration (C_e_) (left) and the corresponding fittings to the Langmuir isotherm model (right) for (**A**) Php, (**B**) BPA, (**C**) PR∙HCl and (**D**) 2-NO. Data represent mean ± S.D. (*n* = 3).

The adsorption isotherms of the different dye and pollutants by the β-CD-PAAm gel fitted well into the Langmuir model with all the correlation coefficient R^2^ ≥ 0.9559 (Figure 8). This indicates the homogeneous distribution of the β-CD units inside the hydrogels, as compared to the other β-CD-based hydrogel system [41]. This might be due to the different preparation methods of the hydrogels. The β-CD-PAAm gel was formed from polymerization and crosslinking from a homogeneous solution. The β-CD units were likely to be distributed evenly inside this network. In addition, the large pore size and the high water content (up to 97%) of β-CD-PAAm gel may make the β-CD units more accessible to the pollutants. The pollutant molecules may diffuse easily into the gel, fit into the hydrophobic β-CD cores and form 1:1 inclusion complexes. According to literatures and various studies regarding the β-CD-based adsorbents [28,38], this type of adsorption by our system should fit into the pseudo-second-order kinetic model and Langmuir isotherm model the best, which was well supported by our good fittings.

After reaching equilibrium, β-CD-PAAm gel which was immersed in 10 mL of 1.0 mM BPA was removed from the solution, lyophilized, and then imaged by SEM to study its surface and cross-sectional morphologies (Figure S4). It was observed that, after adsorption, the β-CD-PAAm gel remained porous, similar to that before adsorption. The large pores may ensure that the β-CD units are easily accessible to the pollutants.

Table 4 summarizes the calculated equilibrium constant K and the q_max,e_ values. The values for BPA adsorption are consistent with the reported values for a β-CD-based hydrogel system [42]. It was observed that the q_max,e_ value was higher for Php and BPA, and was the lowest for PR·HCl. This might be because the association constants between β-CD and Php (3.94 × 10^4^ M^−1^ [26] or 2.80 × 10^4^ M^−1^ [27]) and between β-CD and BPA (3.50 × 10^4^ M^−1^ [22]) are higher, and those between β-CD and PR·HCl (239 M^−1^ [23] or 195 M^−1^ [24]) and between β-CD and 2-NO (699 M^−1^ [25]) are lower. However, q_max,e_ value for 2-NO was higher than expected, probably because, for the C_e_ tested, the q_e_ value was still increasing and has not reached a plateau. Therefore, the predicted q_max,e_ was higher than expected.

### 3.5. Hydrogel Recycling Studies

**Figure 9:**
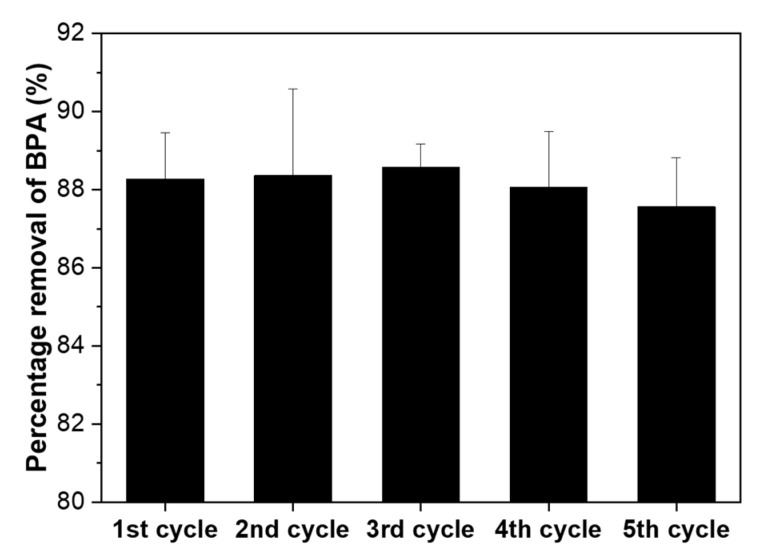
The average percentage removal of BPA by β-CD-PAAm gels for five consecutive adsorption/desorption cycles. Data represent mean ± S.D. (*n* = 3).

Another advantage of β-CD-PAAm gel for pollutant removal is that it can be easily regenerated and reused several times without compromising its performance. To evaluate its recycling properties, the adsorption/desorption cycle was conducted five consecutive times for β-CD-PAAm gel using 0.1 mM BPA solutions. After each adsorption, β-CD-PAAm gel was easily regenerated by soaking in methanol at room temperature for desorption of BPA, before the hydrogel was used again in the next cycle. In Figure 9, it can be observed that the percentage removal of BPA remained almost the same for the five cycles performed, similar to the reported study [28], making the β-CD-PAAm gel more economical and attractive in its applications as an effective and recyclable pollutant removal material.

## 4. Conclusions

β-CD was successfully modified with methacrylate groups by reacting with methacryloyl chloride directly. β-CD-MA with an average substitution degree of 3 methacrylate groups per β-CD was then incorporated into a PAAm hydrogel by copolymerizing with acrylamide monomers via free-radical copolymerization, forming β-CD-PAAm hydrogel. As β-CD-MA with multiple methacrylate groups could act as crosslinkers, no additional crosslinkers, such as MBA crosslinkers, were needed for the gel formation. SEM images revealed that β-CD-PAAm gel had larger pore size than the control PAAm gel, which was synthesized using MBA crosslinkers in the absence of β-CD-MA. The swelling ratio of β-CD-PAAm gel (29.4 g of water/g of hydrogel) was also found to be higher than that of PAAm gel (12.7 g of water/g of hydrogel). Subsequently, the adsorption behaviors of the β-CD-PAAm gel were evaluated using Php as a model dye and BPA, PR·HCl and 2-NO as model pollutants from different classes. In the kinetic adsorption studies, the data for β-CD-PAAm gel fitted well into the pseudo-second-order model for all dye and pollutants, while PAAm gel did not show much adsorption due to the absence of β-CD units. In addition, the β-CD-PAAm gel demonstrated effective adsorption of the different dye and pollutants at various concentrations in the thermodynamic studies. The very low adsorption by PAAm gel further confirmed that the pollutant removal was mainly due to the inclusion complexation ability of β-CD units. The adsorption isotherms by the β-CD-PAAm gel fitted well into the Langmuir model for the different dye and pollutants. Moreover, it was shown that the β-CD-PAAm gel could be easily regenerated by soaking in methanol and reused without losing its adsorption ability for five consecutive adsorption/desorption cycles. Therefore, the easy-to-handle β-CD-PAAm hydrogel platform with effective adsorption properties towards various dye and pollutants shows great potential as a promising pollutant removal system for wastewater treatment applications.

## Figures and Tables

**Table 1:** Surface chemical composition of PAAm gel and β-CD-PAAm gel by XPS.

Sample	Atomic Concentration (%)		
	C	O	N
PAAm gel	65.72	17.11	17.17
β-CD-PAAm gel	64.42	22.01	13.57

**Table 2:** Swelling analysis of PAAm gel and β-CD-PAAm gel **^a^**.

Sample	Weight (mg)		Swelling Ratio
	Swollen (W_s_)	Dry (W_d_)	
PAAm gel	590.1 ± 22.3	43.1 ± 1.0	12.7 ± 0.6
β-CD-PAAm gel	1272.3 ± 45.4	42.0 ± 2.6	29.4 ± 2.4

^a^ After synthesis and purifications, each piece of the disk-shape hydrogels fully swollen in DI water was weighed to get W_s_, and then lyophilized and weighed again to get W_d_. Swelling ratio was calculated from W_s_ and W_d_. Data represent mean ± S.D. (*n* = 5).

**Table 3:** Rate of pollutant removal by β-CD-PAAm gels.

Pollutant	MW	k_2_ (g/mg min)	Correlation Coefficient R^2^	q_e,cal_ (mg/g)	q_e,exp_ (mg/g)
Php	318.32	0.0051	0.9999	6.21	6.07
BPA	228.29	0.0096	0.9999	4.86	4.78
PR·HCl	295.80	0.0266	0.9999	3.84	3.83
2-NO	144.17	0.0203	0.9997	2.32	2.29

**Table 4:** Adsorption equilibrium constant for each pollutant by β-CD-PAAm gels.

Pollutant	K (M^−1^)	Correlation Coefficient R^2^	q_max,e_ (mg/g)
Php	22,000	0.9946	34.97
BPA	19,882	0.9559	29.59
PR·HCl	34,824	0.9673	8.45
2-NO	811	0.9965	55.56

## Data Availability

Data are available from the authors.

## Supplementary Materials

The following are available online. Figures S1–S4: Characterizations of β-CD-MA using ^1^H NMR and FTIR. XPS survey spectra of PAAm gel and β-CD-PAAm gel. SEM images of β-CD-PAAm gel after adsorption of BPA.

## Sample Availability

Samples of the compounds are available from the authors.
