# Supplementary material for: β-Cyclodextrin-Polyacrylamide Hydrogel for Removal of Organic Micropollutants from Water
Source: Molecules. 2021 Aug 19;26(16):5031. doi: 10.3390/molecules26165031 (PMC8402003; doi:10.3390/molecules26165031)
Supplement: Supplementary file 1 [file molecules-26-05031-s001.zip › molecules-1321978-supplementary.pdf]

Supplementary Information

β-Cyclodextrin-Polyacrylamide Hydrogel for Removal of Organic Micropollutants from Water

Xia Song [1,†], Nana Nyarko Mensah [1,2,†], Yuting Wen [1], Jingling Zhu [1], Zhongxing Zhang [1], Wui Siew Tan [2], Xinwei Chen [2] and Jun Li [1,*]

[1] Department of Biomedical Engineering, Faculty of Engineering, National University of Singapore, 7 Engineering Drive 1, Singapore 117574, Singapore; a0045788@u.nus.edu (X.S.); nn.mensah@u.nus.edu (N.N.M.); bieweny@nus.edu.sg (Y.W.); erizhuj@nus.edu.sg (J.Z.); biezhozh@nus.edu.sg (Z.Z.)

[2] Institute of Materials Research and Engineering, A*STAR (Agency for Science, Technology and Research), 2 Fusionopolis Way, Singapore 138634, Singapore; wuisiew@gmail.com (W.S.T.); CHEN_Xinwei@nrf.gov.sg (X.C.)

* Correspondence: jun-li@nus.edu.sg or jun-li@u.nus.edu; Tel. +65-65-167-273

† These authors contribute equally.

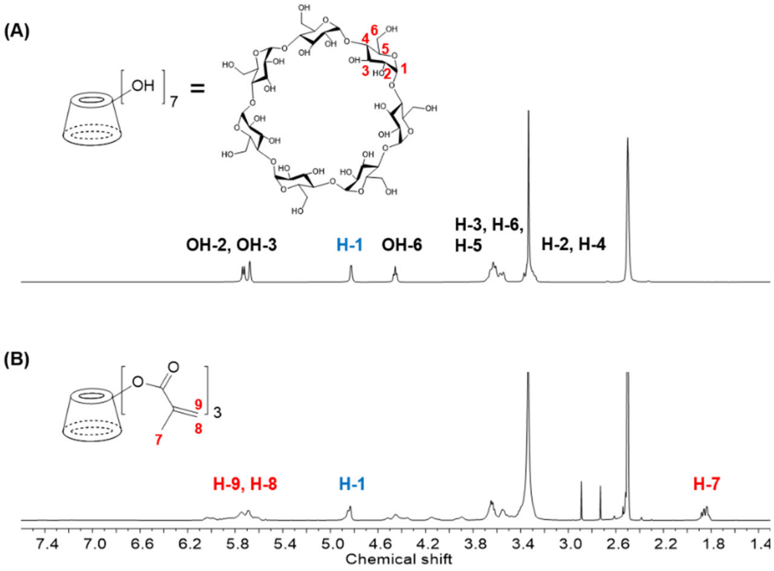

Figure S1. The 600 MHz ^{1}H NMR spectra of (A) pure β-CD and (B) β-CD-MA measured in DMSO-d6 at room temperature.

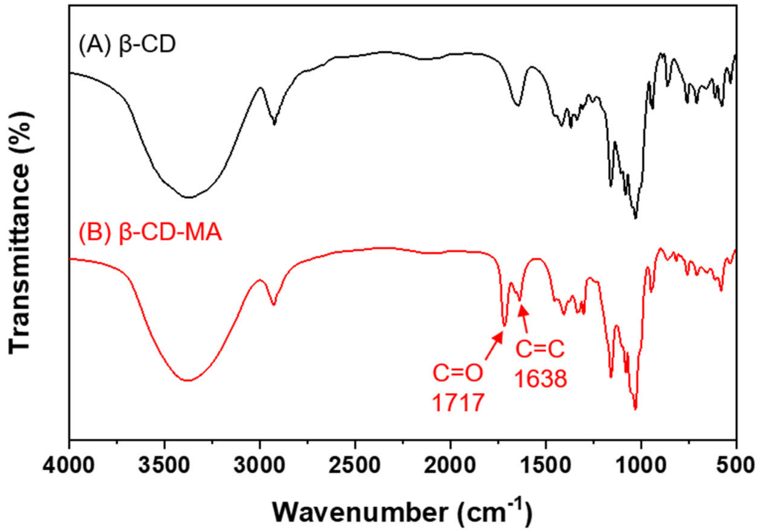

Figure S2. FTIR spectra of (A) β-CD and (B) β-CD-MA.

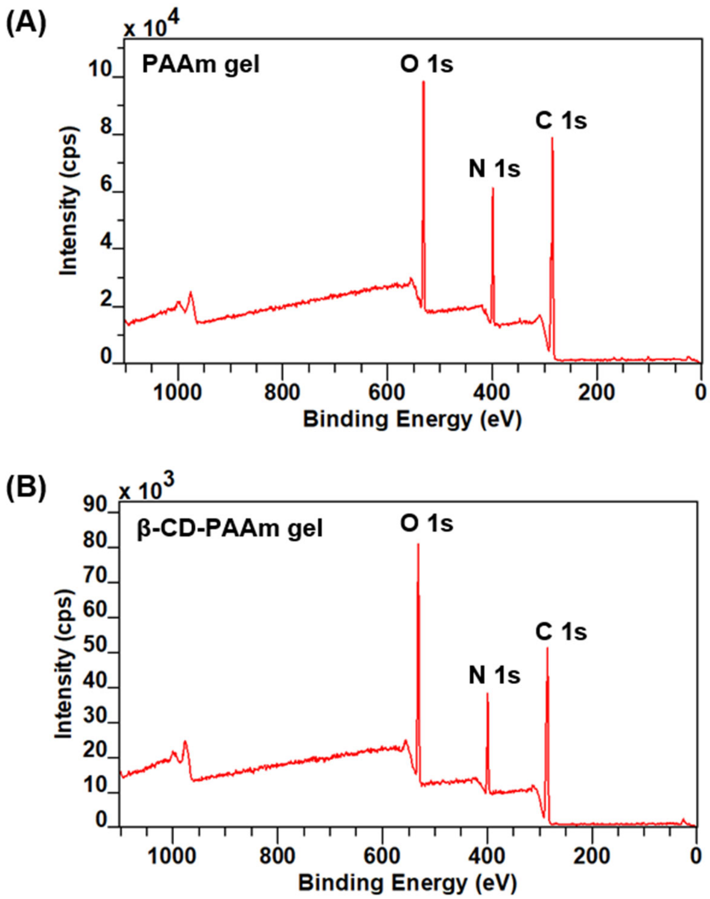

Figure S3. XPS survey spectra of (A) PAAm gel and (B) β-CD-PAAm gel.
